# F. St.John Bullen, M.R.C.S., L.R.C.P.

**Published:** 1917-04

**Authors:** 


### F. ST.JOHN BULLEN, M.R.C.S., L.R.C.P.

A SKILFUL physician and cultured gentleman of a most loveable disposition has passed from our midst by the death at the age of 54 of F. St.John Bullen. He was born at Kennington, the son and grandson of medical practitioners, and was educated at St. Thomas Hospital School, qualifying in the year 1885. After holding a resident post at Evelina Hospital, he was appointed Pathologist and Medical Assistant at Wakefield Asylum, and remained there seven years, during which time he did much original work of high value.

In 1884 he came to practise at Clifton, and married a daughter of Mr. Heber Mardon.

St.John Bullen was a man of many parts. His special knowledge of mental diseases and psychology was of great value to our *Journal,* especially in critical reviews of works on these subjects.

As a musician he had few peers amongst amateurs, and would have done credit to the musical profession had he made that his career. He was a highly skilful pianist and organist, and played the violin and viola. Whilst at Wakefield he conducted the Wakefield Orchestral Society, and composed and arranged for orchestra many works, and in later years at Bristol he assisted at many orchestral concerts.

Architecture, more especially church architecture, claimed his attention, and his knowledge on these subjects and of archæology was extensive.

His health began to fail about two years prior to his death, suffering from progressive muscular atrophy. He bore the steady and pitiless progress of the disease with extraordinary gallantry, and never lost his cheerful disposition even to the last, when the end came suddenly.

He was a man who made friends rather than acquaintances, and enemies never ; truly a man without guile.

